# The Belgica 121 expedition to the Western Antarctic Peninsula: a detailed biodiversity census

**DOI:** 10.3897/BDJ.9.e70590

**Published:** 2021-09-23

**Authors:** Bruno Danis, Henrik Christiansen, Charlène Guillaumot, Franz Maximilian Heindler, Quentin Jossart, Camille Moreau, Francesca Pasotti, Henri Robert, Ben Wallis, Thomas Saucède

**Affiliations:** 1 Université Libre de Bruxelles, Brussels, Belgium Université Libre de Bruxelles Brussels Belgium; 2 KULeuven, Leuven, Belgium KULeuven Leuven Belgium; 3 Vrije Universiteit Brussel, Brussels, Belgium Vrije Universiteit Brussel Brussels Belgium; 4 UGent, Gent, Belgium UGent Gent Belgium; 5 EMC2, Brussels, Belgium EMC2 Brussels Belgium; 6 Ocean Expeditions, Sydney, Australia Ocean Expeditions Sydney Australia; 7 UMR 6282 Biogéosciences, Univ Bourgogne Franche-Comté, CNRS, Dijon, France UMR 6282 Biogéosciences, Univ Bourgogne Franche-Comté, CNRS Dijon France

**Keywords:** Southern Ocean, Belgica, low environmental impact, shallow waters, climate change, benthos

## Abstract

**Background:**

This dataset relates to the biodiversity census carried out during the Belgica 121 (B121) expedition to the Western Antarctic Peninsula from February to March 2019. One of the aims of the campaign was to explore the surroundings of the Gerlache Strait and to carry out a detailed biodiversity census focusing on inter- and subtidal shallow-water areas using both classic descriptive marine ecology methods, as well as state-of-the art techniques (habitat mapping, genetics, trophic ecology). The biodiversity census was carried out onboard a nimble research vessel, RV Australis. This dataset will offer access to the raw data on biodiversity occurrences, obtained using a range of methods described in this data paper.

**New information:**

New raw biodiversity data for a poorly-sampled region (Western Antarctic Peninsula) with a special focus on shallow ecosystems.

## Introduction

Global warming-related environmental changes are expected for large areas of the Southern Ocean in terms of sea ice cover, ocean and glacier melting (Gutt et al. 2015). The Western Antarctic Peninsula (WAP) is one of Earth’s regions where we observe the most rapid and dramatic environmental changes in marine ecosystems, with strong variations in the duration of the sea ice season, extended glacier retreats, ice shelf collapse, warming of surface waters and shifts in local primary production (Ducklow et al. 2013, Stammerjohn et al. 2008, Turner et al. 2014). These climate change related processes are significantly affecting marine ecosystems and their suitability to key-organisms (Carlini et al. 2009, Clarke et al. 2007, Constable et al. 2014, Sahade et al. 2015, Pasotti et al. 2015). Furthermore, recent efforts in documenting the biodiversity of the Southern Ocean has shown that intensity in biological sampling has considerably varied between Antarctic regions and time periods since first expeditions were carried out (Griffiths et al. 2011, De Broyer et al. 2014). Most data in the distribution of sampling intensity, including animal tagging and watching, are from locations nearby national scientific bases and along main transit routes of research vessels that regularly visit these bases, mostly for logistic reasons (Griffiths 2010).

The WAP is a sea ice dependent ecosystem which is experiencing rapid, transition-inducing environmental changes (Ducklow et al. 2013, Turner et al. 2014). In a comprehensive synthesis paper, Gutt et al. (2015) showed that the spatial scale of past changes in sea ice extent is larger than that of ocean warming. The response of marine organisms and ecosystem processes to such environmental changes is still poorly understood (Siegert et al. 2019). Yet the available studies show, in general, a high sensitivity of these highly adapted species and hint to a vulnerability of the ecological processes that they mediate (Ducklow et al. 2013, Chown et al. 2015 and references therein). Insights on resilience, thresholds and tipping points for species, communities and ecosystems are, therefore, of paramount importance to the understanding of ongoing large-scale changes (Convey et al. 2014, Kennicutt et al. 2015, Oliver et al. 2015). Recent studies have shown that WAP fjord basins exhibited 3 to 38-fold greater benthic megafaunal abundance than the open shelf and that local species diversity and trophic network complexity remained high from outer to inner fjord basins (Grange and Smith 2013), even if our current knowledge of faunal biodiversity is considered as patchy and incomplete (Friedlander et al. 2020). As WAP fjords also provide important habitat and foraging areas for Antarctic krill and Baleen whales, there is an urgent need to develop better understanding of the structure, dynamics and climate-sensitivity of WAP subpolar fjord ecosystems (Grange and Smith 2013).

In February 2019, the Belgica 121 expedition (B121) aimed to fill knowledge gaps in this potential biodiversity hotspot (whilst limiting its environmental footprint) by making use of a nimble sampling platform, the RV Australis. The RV Australis is a steel-hulled, rugged motor sailing vessel which carries a comprehensive range of safety, operational and navigational equipment. B121 sampled a broad area along the northern coast of the WAP, extending from the Berthelot Islands to the SW to Enterprise Islands to the NE and including a total of 15 stations selected for their contrasting conditions in terms of sea ice dynamics, glacier activity, biodiversity and oceanographic conditions and pressure by human visitors. This data paper relates to the biodiversity census carried out during the B121 expedition (for the full report, see Danis et al. 2019).

## Project description

### Title

The Belgica 121 expedition to the Western Antarctic Peninsula: a high resolution biodiversity census

### Personnel

Bruno Danis

### Study area description

The study area was primarily the Western Antarctic Peninsula in the Southern Ocean. B121 took place between February and March 2019, sampling 15 stations in 22 working days in an area extending from Berthelot (65°19.751 S, 64°08.263 W) to Enterprise (64°32.420 S, 61°59.899 W) Islands.

### Design description

The overarching objective of the expedition was to gather samples and data to help build a benchmark to better understand the response of shallow benthic communities to variable glacial regimes in a fast-warming region of the Southern Ocean, the WAP. The collected samples are expected to help refine insights gained in the plasticity/resilience of these communities in the framework of the RECTO/vERSO projects (http://rectoversoprojects.be). The objective was tackled by using a multi-faceted approach, matched by the complementary competences of the scientific crew and sampling gear. The expedition was a unique opportunity to address a series of underlying scientific/logistic questions. Amongst these questions, the expedition focused on testing the concept of using a nimble platform for Antarctic marine biology fieldwork and its potential to fill knowledge gaps with a limited environmental impact, mapping the marine habitats in selected locations of the Gerlache Strait and assessing different biodiversity levels in various locations of the WAP, from the supratidal to 20 m depth.

### Funding

The Belgian Science Policy Office (BELSPO): the bulk of the funding of the expedition was channelled through two research projects funded by BELSPO, RECTO (promoter: Isa Schön, Royal Belgian Institute of Natural Sciences) and vERSO (promoter: Bruno Danis, Université Libre de Bruxelles). The Cabinet Marcourt (Federation Wallonia-Brussels – Research, Education) supported the expedition for functioning and various equipment. The Belgian Federal Public Service Health, Food Chain Safety and Environment funded the ship time necessary to the visit of historic monument N°45 dedicated to the Belgica expedition, led by Adrien de Gerlache. The Fund for Scientific Research – FNRS and the Research Foundation – Flanders (FWO) have funded travel expenses. The B121 team also acknowledges financial support from the Fonds Léopold III and the Royal Belgian Zoological Society.

## Sampling methods

### Study extent

The expedition took place between 23 February and 24 March 2019. RV Australis departed from Ushuaia (Argentina) on February 23 and arrived at the first sampling station (Melchior Islands) on 27 February after crossing the Drake Passage. The last station was completed on 20 March and the expedition returned to Ushuaia on 24 March, a total of 22 days being devoted to the sampling effort, including bird and marine mammal observations. The sampling area focused on the WAP and extended from the Berthelot Islands to the SW to Enterprise Islands to the NE and included a total of 15 stations. Nearly half the stations were exhaustively sampled according to the initial protocol established (see Table 1, in bold), while others were partially worked out as timing, priorities, anchoring and weather allowed. Metchnikoff Point (MP) was visited in order to check the status of historic monument #45 at the request of the Belgian Federal Service Food, Health and Environment Foundation.

### Sampling description

The expedition aimed to focus on carrying out a detailed biodiversity census of shallow areas, from the intertidal to the subtidal zones (up to 20 m depth) at 15 stations within the Gerlache Strait. The stations were chosen for their contrasting conditions in terms of exposure to glaciers influence, iceberg scouring, to ocean water masses and currents (Drake Passage, Gerlache Strait etc.), geomorphology, penguins colonies and direct anthropogenic stressors (tourism and maritime traffic). Multiple types of gear were deployed (see Table 2), combining traditional marine ecology instruments (traps, nets, grabs, ...) and modern techniques (drones, ROVs). The team was mostly composed of young scientists who were acquainted with the use of several techniques. Each team had a specific project and was able to help others during sample processing stages. The initial stages of the expedition were exploratory (one full station would need up to 4 days to be completed) and were followed by more efficient sampling (1.5–2 days per station). Opportunistically, certain stations were partially sampled as a function of priorities and weather/anchoring conditions.

### Quality control

In the framework of the B121 expedition, data were aggregated and organised to ensure optimal use in the future for data publication in authoritative repositories and sample management. A series of data types were collected pertaining to navigation, weather conditions and sampling efforts (both biological and oceanographic). General procedures: Logbooks: hard copies of logbooks were completed on a daily basis by the B121 team. Data were organised in four different logbooks: sample, events, photo and diving. Logbooks were digitised and backed up on a daily basis. Spreadsheets: data from the logbooks were entered in a dedicated spreadsheet on a daily basis by two members of the B121 team: Charlène Guillaumot and Bruno Danis. Quality control (QC) was performed on the fly and feedback was given to the researchers on an ad hoc basis. Backup procedures: digital data and samples were backed up on a daily basis on two computers and two external hard drives. Sample (biodiversity) data: Sample data were gathered in MS Excel spreadsheets, specially prepared for the expedition. The structure of the spreadsheet is based upon the Darwin Core (DwC) standard, expanded for specific data and sample management needs. A template of this spreadsheet is provided in an annex for future use by other users. Identifications were carried out in the field and taxonomic data were cross-checked against the content of the World Register of Marine Species Taxon Match tool (http://www.marinespecies.org/aphia.php?p=match). For specimens we were not able to identify, help is sought from taxonomic experts and the dataset will be updated accordingly. Media data: Large amounts of video data were gathered in the framework of the expedition, both for outreach and research purposes. Underwater footage was taken by Bruno Danis and Henri Robert using a Remotely Operated Vehicle (ROV: OpenROV Trident). The footage was used essentially for exploration and dive site confirmation purposes. Aerial footage was shot by Franz Heindler, Camille Moreau and Bruno Danis using two DJI Mavic Pro drones, for documentation purposes. Macrophotography of the most common species was carried out by Quentin Jossart. Documentary footage was mostly shot by Franz Heindler and other members of the team. For more details, see the dedicated section below. Data publication: In the spirit of the Antarctic Treaty, Art. 3.1.c, the data emerging from the Belgica 121 sampling efforts will be made openly and freely available, in the best possible time limits and will follow the standards, policies and norms of behaviour as established by the Scientific Committee on Antarctic Research (SCAR). In particular, raw biodiversity data will be shared using dedicated, community-driven platforms, such as the biodiversity.aq initiative. Processed data will be made available through scientific publications and through the Belgica 121 website (www.belgica121.be).

### Step description

Full description of methodologies is available from the B121 expedition report (Danis et al. 2019): http://belgica120.be/wp-content/uploads/2019/05/B121-Cruise-report.pdf. Briefly, for the macro and mega benthos survey, the diversity analysis was conducted using various sampling gears and investigation means as a necessary preliminary step to further ecological analyses, from individual species systematics to trophic and community analyses. Most common and key species (engineers or top predators) of the surveyed shallow water habitats (between 5 and 20 m depth) could be observed and identified during the dives, some of them sampled by hand picking or identified on video transects. This first inventory was widely complemented by samples collected with a Rauschert dredge, Van Veen grab and amphipod trap. For the soft sediment biodiversity, samples for meiofauna assemblage structure (taxa diversity, nematode diversity, biomass), were collected at each location by divers either by means of perspex push cores (3.6 cm diameter, quantitative) or by surface sediment scooping (qualitative). Where the sediment characteristics allowed core sampling, the sediment was sliced in different layer profiles (0–1 cm, 1–2 cm, 2–5 cm, 5–10 cm) for the whole core depth. At least three replicates were taken for the meiofauna characterisation at each location dive event. For the intertidal work, two sampling procedures were used to characterise the biodiversity and abundance on each site: (1) 10 quadrats (25 cm × 25 cm) were randomly disposed at the low tide level. Presence and abundance of each species (morphotypes) were recorded within each quadrat and specimens were preserved in 96% ethanol for further identification and analyses; (2) to obtain a better overview of the total biodiversity, an exploration (1 hour) in the vicinity of the quadrats was also done to look for any species not found inside the quadrats. Fish biodiversity was addressed using three methods: (1) angling with hooks, line and sinker, (2) gill nets and (3) a cylindrical fish trap or fyke. Angling took place with standard commercial fishing rods, braided fishing line and rigs (Sabikis), equipped with multiple hooks of varying sizes and small, colourful lures, luminescent plastic beads and weights at the end in depths of 5–50 m. Hooks were sometimes baited with fish, mollusc or shrimp and used actively (jigging during daytime from the ship or zodiacs) or passively (fixed to the ship overnight). Two types of gill nets were used, measuring approximately 18 m in width and 1.5 m in height and with 4 cm and 8 cm mesh size (stretched), respectively. Nets were set in depths of 10–30 m and usually perpendicular to observed currents. The fish trap was deployed for at least 8 h in depths of 10–30 m, baited with fish, molluscs or shrimp. Finally, continuous monitoring of birds and marine mammals (species identification and headcount) was performed from the bridge or a spot offering the best visibility on deck. Bird/mammal standard counts are 30 min non-stop observation with binoculars for identification (if required) and age/sex determination when possible. A 300 mm telephoto lens was used for documentation and identification of species that pose identification issues in the field (e.g. *Catharacta* spp., *Pachyptila* spp.). GPS ship position and climatic conditions were recorded at each start and end position of counts. Counts were performed during daylight (from dawn to dusk) and only during good visibility (counts must be stopped when visibility is poor due to heavy fog or precipitation) to avoid bias in animal detection and subsequent false population estimates.

## Geographic coverage

### Description

**Figure 1.**
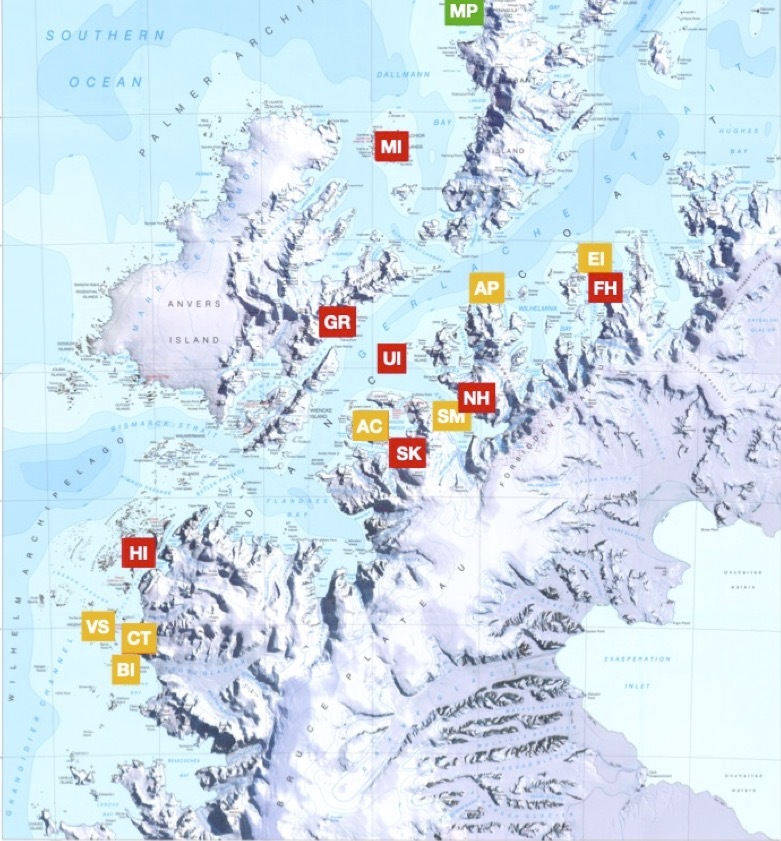
General map of the sampling area. Red rectangles: complete stations; orange rectangles: partial stations; green rectangle: historic monument visit. Modified after MAP “Brabant Islands to Argentine Islands”, British Antarctic Survey, Edition 1, 2008.

The sampling area focused on the Western Antarctic Peninsula (WAP) and extended from Berthelot Island to the SW to Enterprise Island to the NE and included a total of 15 stations (see Fig. 1). Certain stations were exhaustively sampled, while others were partially worked out as timing, priorities, anchoring and weather allowed. Metchnikoff Point (MP) was visited in order to check the status of historic monument #45. The birds and marine mammals survey was carried out all along the expedition and includes the whole expedition track, from Ushuaia (AR) to the WAP.

### Coordinates

-66 and -54 Latitude; -68 and -62 Longitude.

## Taxonomic coverage

### Description

Specimens were collected in the intertidal and subtidal zones (max depth: 20 m). Meiobenthos and megabenthos classes were analysed in particular. Identification of specimens is still ongoing, combining morphological analyses by expert taxonomists and a genetic approach where possible.

### Taxa included

**Table:** 

Rank	Scientific Name	
genus	* Abyssorchomene *	
genus	* Acodontaster *	
genus	* Aequiyoldia *	
species	* Aequiyoldia eightsii *	
order	Amphipoda	
order	Actinari	
species	* Antarctomysis maxima *	
species	* Aphrodroma brevirostris *	
species	* Arctocephalus australis *	
species	* Arctocephalus gazella *	
class	Asteroidea	
species	* Balaenoptera bonaerensis *	
family	Bathydraconidae	
class	Bivalvia	
phylum	Bryozoa	
genus	* Candelabrum *	
genus	* Catharacta *	
species	* Chaenocephalus aceratus *	
species	* Charcotia obesa *	
species	* Chionis albus *	
class	Polyplacophora	
phylum	Chlorophyta	
phylum	Cnidaria	
class	Collembola	
subphylum	Crustacea	
species	* Cuenotaster involutus *	
order	Cumacea	
species	* Daption capense *	
order	Decapoda	
genus	* Dendrilla *	
genus	* Desmarestia *	
species	* Desmarestia antarctica *	
species	* Diomedea exulans *	
species	* Diplasterias brucei *	
genus	* Doris *	
phylum	Echinodermata	
class	Eucarida	
genus	* Euneognathia *	
order	Euphausiacea	
genus	* Eusirus *	
genus	* Flabelligera *	
species	* Fregetta tropica *	
species	* Fulmarus glacialoides *	
class	Gastropoda	
species	* Glyphoperidium bursa *	
genus	* Glyptonotus *	
species	* Glyptonotus antarcticus *	
species	* Gobionotothen gibberifrons *	
genus	* Granaster *	
species	* Granaster nutrix *	
species	* Halobaena caerulea *	
genus	* Harpagifer *	
species	* Harpagifer antarcticus *	
genus	* Himantothallus *	
class	Holothuroidea	
species	* Homaxinella balfourensis *	
species	* Hydrurga leptonyx *	
order	Isopoda	
genus	* Labidiaster *	
species	* Lagenorhynchus australis *	
species	* Lagenorhynchus cruciger *	
species	* Larus dominicanus *	
species	* Laternula elliptica *	
species	* Leptonychotes weddellii *	
species	* Lindbergichthys nudifrons *	
species	* Lobodon carcinophagus *	
genus	* Lysasterias *	
order	Lysianassoidea	
species	* Macronectes giganteus *	
genus	* Margarella *	
species	* Margarella antarctic *	
species	* Megaptera novaeangliae *	
phylum	Mollusca	
genus	* Mycale *	
species	Mycale (Oxymycale) acerata	
order	Mysida	
species	* Nacella concinna *	
phylum	Nematoda	
phylum	Nemertea	
species	* Neosmilaster georgianus *	
species	* Notothenia coriiceps *	
species	* Notothenia rossii *	
order	Nudibranchia	
species	* Oceanites oceanicus *	
genus	* Odontaster *	
species	* Odontaster meridionalis *	
species	* Odontaster pearsei *	
species	* Odontaster roseus *	
species	* Odontaster validus *	
genus	* Ophionotus *	
species	* Ophionotus victoriae *	
class	Ophiuroidea	
class	Ostracoda	
species	* Otaria byronia *	
genus	* Pachyptila *	
species	* Pachyptila desolata *	
species	* Pagodroma nivea *	
genus	* Parborlasia *	
species	* Parborlasia corrugatus *	
species	* Pelecanoides urinatrix *	
genus	* Perknaster *	
species	* Phalacrocorax atriceps *	
species	* Phoebetria palpebrata *	
order	Pinnipedia	
class	Polychaeta	
class	Polyplacophora	
phylum	Porifera	
species	* Procellaria aequinoctialis *	
species	* Procellaria cinerea *	
species	* Pseudorchomene plebs *	
species	* Psilaster charcoti *	
species	* Pterodroma mollis *	
species	* Puffinus griseus *	
class	Pycnogonida	
genus	* Pygoscelis *	
species	* Pygoscelis adeliae *	
species	* Pygoscelis antarcticus *	
species	* Pygoscelis papua *	
order	Sphenisciformes	
species	* Spheniscus magellanicus *	
genus	* Sphyraena *	
species	* Staurocucumis turqueti *	
species	* Stercorarius chilensis *	
species	* Stercorarius maccormicki *	
species	* Sterechinus neumayeri *	
species	* Sterna hirundinacea *	
order	Tanaidacea	
species	* Thalassarche chrysostoma *	
species	* Thalassarche melanophris *	
species	* Thalassoica antarctica *	
species	* Trematocarpus antarcticus *	
species	* Trematomus bernacchii *	
species	* Trematomus newnesi *	
subphylum	Tunicata	
class	Echinoidea	
species	* Sterechinus neumayeri *	

## Traits coverage

### Data coverage of traits

PLEASE FILL IN TRAIT INFORMATION HERE

## Temporal coverage

**Data range:** 2019-2-23 – 2019-3-24.

## Collection data

### Collection name

B121 expedition collection, hosted at the BIOMAR Lab, Université Libre de Bruxelles

### Specimen preservation method

ethanol, deep frozen, RNA later, other

## Usage licence

### Usage licence

Creative Commons Public Domain Waiver (CC-Zero)

### IP rights notes

This work is licensed under a Creative Commons Attribution (CC-BY) 4.0 Licence.

## Data resources

### Data package title

The Belgica 121 expedition to the Western Antarctic Peninsula: a high resolution biodiversity census

### Resource link

https://www.gbif.org/dataset/b635be2e-76ea-4600-8f83-549601653c0a

### Number of data sets

1

### Data set 1.

#### Data set name

The Belgica 121 expedition to the Western Antarctic Peninsula: a high resolution biodiversity census

#### Data format

Darwin Core

#### Number of columns

43

#### Character set

UTF-8

#### Description

This dataset (Danis 2021) pertains to the outputs of the Belgica 121 (B121) expedition, whose aim was to explore the surroundings of the Gerlache Strait (Western Antarctic Peninsula) and to carry out a detailed biodiversity census focusing on intertidal and shallow areas using both classic descriptive marine ecology methods as well as state-of-the-art techniques (habitat mapping, genetics, trophic ecology). This dataset will offer access to the raw data on biodiversity occurrences, obtained using a range of methods.

**Data set 1.** 

Column label	Column description
datasetID	An identifier for the set of data. May be a global unique identifier or an identifier specific to a collection or institution.
occurrenceID	An identifier for the Occurrence (as opposed to a particular digital record of the occurrence). In the absence of a persistent global unique identifier, construct one from a combination of identifiers in the record that will most closely make the occurrenceID globally unique.
eventID	An identifier for the set of information associated with an Event (something that occurs at a place and time). May be a global unique identifier or an identifier specific to the dataset.
recordNumber	An identifier given to the Occurrence at the time it was recorded. Often serves as a link between field notes and an Occurrence record, such as a specimen collector's number.
eventDate	The date-time or interval during which an Event occurred. For occurrences, this is the date-time when the event was recorded. Not suitable for a time in a geological context.
year	The four-digit year in which the Event occurred, according to the Common Era Calendar.
month	The integer month in which the Event occurred.
day	The integer day of the month on which the Event occurred.
eventTime	The time or interval during which an Event occurred.
vernacularName	A common or vernacular name.
scientificName	The full scientific name, with authorship and date information, if known. When forming part of an Identification, this should be the name in the lowest level taxonomic rank that can be determined. This term should not contain identification qualifications, which should instead be supplied in the IdentificationQualifier term.
occurrenceStatus	A statement about the presence or absence of a Taxon at a Location.
institutionID	An identifier for the institution having custody of the object(s) or information referred to in the record.
basisOfRecord	The specific nature of the data record.
individualCount	The number of individuals represented present at the time of the Occurrence.
footprintWKT	A Well-Known Text (WKT) representation of the shape (footprint, geometry) that defines the Location. A Location may have both a point-radius representation (see decimalLatitude) and a footprint representation and they may differ from each other.
decimalLatitude	The geographic latitude (in decimal degrees, using the spatial reference system given in geodeticDatum) of the geographic centre of a Location. Positive values are north of the Equator, negative values are south of it. Legal values lie between -90 and 90, inclusive.
decimalLongitude	The geographic longitude (in decimal degrees, using the spatial reference system given in geodeticDatum) of the geographic centre of a Location. Positive values are east of the Greenwich Meridian, negative values are west of it. Legal values lie between -180 and 180, inclusive.
coordinatePrecision	The horizontal distance (in metres) from the given decimalLatitude and decimalLongitude describing the smallest circle containing the whole of the Location. Leave the value empty if the uncertainty is unknown, cannot be estimated or is not applicable (because there are no coordinates). Zero is not a valid value for this term.
occurrenceRemarks	Comments or notes about the Occurrence.
genus	The full scientific name of the genus in which the taxon is classified.
specificEpithet	The name of the first or species epithet of the scientificName.
identifiedBy	A list (concatenated and separated) of names of people, groups or organisations who assigned the Taxon to the subject.
recordedBy	A list (concatenated and separated) of names of people, groups or organisations responsible for recording the original Occurrence. The primary collector or observer, especially one who applies a personal identifier (recordNumber), should be listed first.
preparations	A list (concatenated and separated) of preparations and preservation methods for a specimen.
dynamicProperties	A list of additional measurements, facts, characteristics or assertions about the record. Meant to provide a mechanism for structured content.
eventRemarks	Comments or notes about the Event.
locality	The specific description of the place. Less specific geographic information can be provided in other geographic terms (higherGeography, continent, country, stateProvince, county, municipality, waterBody, island, islandGroup). This term may contain information modified from the original to correct perceived errors or standardise the description.
maximumDepthInMetres	The greater depth of a range of depth below the local surface, in metres.
minimumDepthInMetres	The lesser depth of a range of depth below the local surface, in metres.
modified	The most recent date-time on which the resource was changed.
parentEventID	An identifier for the broader Event that groups this and potentially other Events.
samplingProtocol	The name of, reference to, or description of the method or protocol used during an Event.
type	The nature or genre of the resource.
waterbody	The name of the water body in which the Location occurs.
class	The full scientific name of the class in which the taxon is classified.
family	The full scientific name of the family in which the taxon is classified.
fieldNumber	An identifier given to the event in the field. Often serves as a link between field notes and the Event.
identificationQualifier	A brief phrase or a standard term ("cf.", "aff.") to express the determiner's doubts about the Identification.
kingdom	The full scientific name of the kingdom in which the taxon is classified.
phylum	The full scientific name of the phylum or division in which the taxon is classified.
order	The full scientific name of the order in which the taxon is classified.
scientificNameID	An identifier for the nomenclatural (not taxonomic) details of a scientific name.

## Figures and Tables

**Table 1.** Station list including location and sampling dates. Fully sampled stations are in bold.

**Stations**		**Lat (S)**	**Long (W)**	**Arrival**	**Departure**
**MI**	**Melchior Island**	**64°19.246**	**62°55.375**	**27/02/2019**	**03/03/2019**
MP	Metchnikoff Point	64°02.395	62°34.078	03/03/2019	03/03/2019
**NH**	**Nekko Harbor**	**64°50.565**	**62°32.009**	**03/03/2019**	**06/03/2019**
SM	SeaMount	64°51.283	62°36.136	06/03/2019	06/03/2019
**UI**	**Useful Island**	**64°43.146**	**62°52.159**	**06/03/2019**	**08/03/2019**
**SK**	**Skontorp Cove**	**64°54.190**	**62°51.845**	**08/03/2019**	**10/03/2019**
AC	Alvaro Cove	64°52.206	63°00.054	10/03/2019	11/03/2019
**HI**	**Hovgaard Islands**	**65°06.057**	**64°04.992**	**11/03/2019**	**13/03/2019**
BI	Berthelot Islands	65°19.751	64°08.263	14/03/2019	14/03/2019
VS	Vernadsky Station	65°14.746	64°15.420	14/03/2019	15/03/2019
CT	Cape Tuxen	64°46.765	63°40.381	15/03/2019	15/03/2019
**GR**	**Green Reef**	**64°43.590**	**63°16.974**	**15/03/2019**	**17/03/2019**
AP	Arctowski Peninsula	64°35.362	62°31.400	18/03/2019	18/03/2019
**FH**	**Foyn Harbour**	**64°32.798**	**61°59.885**	**18/03/2019**	**20/03/2019**
EI	Enterprise Islands	64°32.420	61°59.899	20/03/2019	20/03/2019

**Table 2.** Types of gear deployed during the B121 expedition.

**Code**	**Full name**
AT	Amphipod trap
BN	Bongo net
CTD	CTD
DIV	Scuba divers
DR	Drone
GN	Gillnet
ITD	Intertidal sampling
KELP	Kelp survey
LF	Line fishing
LL	Long line fishing
NIS	Niskin bottle
RD	Rauschert dredge
ROV	Remotely operated vehicle
SP	Snow petrel (hand collecting of feathers)
TER	Terrestrial survey
TOP	Top predator survey
VV	Van Veen grab
